# A molecular survey of vector-borne pathogens in red foxes (*Vulpes vulpes*) from Bosnia and Herzegovina

**DOI:** 10.1186/s13071-015-0692-x

**Published:** 2015-02-08

**Authors:** Adnan Hodžić, Amer Alić, Hans-Peter Fuehrer, Josef Harl, Walpurga Wille-Piazzai, Georg Gerhard Duscher

**Affiliations:** Institute of Parasitology, Department for Pathobiology, University of Veterinary Medicine Vienna, Vienna, Austria; Department of Pathology, Veterinary Faculty, University of Sarajevo, Sarajevo, Bosnia and Herzegovina

**Keywords:** *Babesia* cf. *microti*, *Hepatozoon canis*, *Babesia canis*, Red fox, *Vulpes vulpes*, Bosnia and Herzegovina, PCR

## Abstract

**Background:**

Red foxes (*Vulpes vulpes*) have recently been recognized as potential reservoirs of several vector-borne pathogens and a source of infection for domestic dogs and humans, mostly due to their close vicinity to urban areas and frequent exposure to different arthropod vectors. The aim of this study was to investigate the presence and distribution of *Babesia* spp., *Hepatozoon canis*, *Anaplasma* spp., *Bartonella* spp., ‘*Candidatus* Neoehrlichia mikurensis’, *Ehrlichia canis*, *Rickettsia* spp. and blood filaroid nematodes in free-ranging red foxes from Bosnia and Herzegovina.

**Methods:**

Spleen samples from a total of 119 red foxes, shot during the hunting season between October 2013 and April 2014 throughout Bosnia and Herzegovina, were examined for the presence of blood vector-borne pathogens by conventional PCRs and sequencing.

**Results:**

In the present study, three species of apicomplexan parasites were molecularly identified in 73 red foxes from the entire sample area, with an overall prevalence of 60.8%. The DNA of *B. canis*, *B.* cf. *microti* and *H. canis* was found in 1 (0.8%), 38 (31.9%) and 46 (38.6%) spleen samples, respectively. In 11 samples (9.2%) co-infections with *B.* cf. *microti* and *H. canis* were detected and one fox harboured all three parasites (0.8%). There were no statistically significant differences between geographical region, sex or age of the host in the infection prevalence of *B.* cf. *microti*, although females (52.9%; 18/34) were significantly more infected with *H. canis* than males (32.9%; 28/85). The presence of vector-borne bacteria and filaroid nematodes was not detected in our study.

**Conclusion:**

This is the first report of *B. canis*, *B.* cf. *microti* and *H. canis* parasites in foxes from Bosnia and Herzegovina and the data presented here provide a first insight into the distribution of these pathogens among the red fox population. Moreover, the relatively high prevalence of *B.* cf. *microti* and *H. canis* reinforces the assumption that this wild canid species might be a possible reservoir and source of infection for domestic dogs.

## Background

Vector-borne diseases (VBDs) are caused by many protozoan, helminthic, bacterial and viral pathogens, which are transmitted to animals and humans by blood-sucking arthropods, such as ticks, mosquitos, fleas and phlebotomine sand flies [1]. The majority of VBDs are classified as emerging infectious diseases and anthropogenic changes, such as global warming, deforestation, globalization and pollution, may have an impact on their prevalence and distribution [1,2]. However, despite intensive clinical and epidemiological research in the recent past, especially in domestic dogs and cats, the information on the occurrence and prevalence of vector-borne pathogens in wild canids is still scarce [3-7]. Red foxes (*Vulpes vulpes*) are the most abundant wild canid species in Europe [8] and recently they have been recognized as a potential reservoir for blood vector-borne pathogens, such as *Babesia* spp. [6,9], *Hepatozoon canis* [4,10,11], *Anaplasma phagocytophilum* [12-15], *Bartonella* spp. [16,17], *Ehrlichia canis* [18] and filaroid nematodes [19-21]. They represent an excellent sentinel species and a possible source of several VBDs to domestic animals and humans, mostly due to their close proximity to urban or agricultural areas and frequent exposure to different arthropod vectors [2,6,15,22,23].

Tick-borne parasitic hematozoa of the genus *Babesia* (order Piroplasmida) infect erythrocytes of a wide range of domestic and wild animals [6,9,24]. In the past, it was assumed that only *B. canis* and *B. gibsoni* can cause diseases in dogs [9]. However, a piroplasm closely related to zoonotic *B. microti* (denominated as *B.* cf. *microti*, *B. microti*-like, *B. annae* or *Theileria annae*) was detected from dogs with clinical signs of hemolytic anemia, azotemia and renal failure [25-28]. Recently, *B.* cf. *microti* parasites were also molecularly confirmed in red foxes from Austria [29], Croatia [3], Germany [9], Italy [7], Poland [4], Portugal [6] and Spain [30]. Furthermore, *B. canis* and *B. gibsoni* were described in foxes based on morphological characteristics [24], and the first molecular report of *B. canis* was described in a single fox from Portugal [6].

*Hepatozoon canis* (order Eucoccidiorida) is an apicomplexan protozoan parasite infecting domestic dogs and wild canids worldwide [10,31]. The main vector of *H. canis* is the brown dog tick, *Rhipicephalus sanguineus*, and the pathogen occurrence is mostly related to the geographical distribution of the tick host [32]. Transmission to the vertebrate host typically takes place by ingestion of a tick containing mature oocysts [33], although vertical transmission of the parasite from female foxes to the progeny might also occur [34]. The infection in dogs is often subclinical, but it could be manifested as a severe life-threatening disease with fever, cachexia, lethargy and anemia [35]. In foxes, *H. canis* is highly prevalent and it has been recorded in Austria [29], Croatia [3], Germany [36], Hungary [31], Poland [4], Portugal [10], Romania [11], Slovakia [37] and Spain [38].

In recent years, the interest of the scientific community in vector-borne bacteria from the genera *Anaplasma*, *Bartonella*, *Ehrlichia*, *Rickettsia* and the recently described cluster ‘*Candidatus* Neoehrlichia’ is growing worldwide since they were recognized as important human and animal pathogens. Thus *A. phagocytophilum*, *A. ovis*, *A. bovis*, *B. rochalimae* and *E. canis* were molecularly identified in foxes from many European countries [12,13,15-17,23,39]. Moreover, ‘*Candidatus* N. mikurensis’ and *Rickettsia* spp. were found in humans, domestic and wild animals, and arthropods collected from foxes [13,23,40-42], but they have never been molecularly confirmed in that wild canid species itself.

Canine dirofilariosis, caused by *Dirofilaria immitis* and *D. repens*, was diagnosed in Bosnia and Herzegovina for the first time in 2009, with the prevalence of 3.1% and 1.9% in dogs, respectively [43]. These nematodes infect mainly dogs, but also wild carnivores, cats and humans [20]. Several studies have shown that foxes represent an important wild reservoir for filaroid nematodes (e.g., *Dirofilaria*, *Acanthocheilonema*) and in fact they support the circulation and transmission of microfilariae to companion animals and humans [20,21,44].

To the best of our knowledge, there is no information on vector-borne pathogens in the red fox population from Bosnia and Herzegovina. Therefore, the aim of this study was to investigate the presence and distribution of *Babesia* spp., *Hepatozoon canis*, *Anaplasma* spp., *Bartonella* spp., ‘*Candidatus* N. mikurensis’, *Ehrlichia canis*, *Rickettsia* spp. and blood filaroid nematodes in free-ranging red foxes from Bosnia and Herzegovina.

## Methods

### Collection of samples

The present study was conducted in Bosnia and Herzegovina, which covers 51,209 km^2^ and is located in the western part of the Balkan Peninsula (43° 52’ N, 18° 25’ E). A total of 119 red foxes (85 males, 34 females; 7 juveniles <1 yr., 112 adults >1 yr.) from 29 municipalities of six different regions were shot during the hunting season between October 2013 and April 2014. All animals were immediately delivered to the Department of Pathology at the Veterinary Faculty in Sarajevo and stored at 4°C for no more than 72 h. Data on sex, age and area of origin was recorded for each individual animal. During necropsy, small pieces of spleen tissue were collected, stored at −20°C and sent to the Institute of Parasitology at the University of Veterinary Medicine in Vienna, Austria for further analysis. Additionally, hearts, pulmonary arteries and lungs were dissected and examined visually for the presence of *D. immitis*.

### DNA extraction, PCR amplification and sequencing

Total DNA was extracted from up to 20 mg of spleen tissue using the High Pure PCR Template Preparation Kit (Roche Diagnostics, Germany) according to the manufacturer’s instructions. The PCR primers (Table 1) and amplification conditions for molecular detection of *Babesia* spp., *Anaplasma* spp., *Bartonella* spp., ‘*Candidatus* N. mikurensis’, *E. canis* and *Rickettsia* spp. have been published elsewhere [45-48]. PCR products were separated by electrophoresis in 2% agarose gels stained with Midori Green Advance DNA stain (Nippon Genetics Europe, Germany). All positive samples were purified and directly sequenced by a commercial company (LGC Genomics, Germany). Obtained sequences were edited using the software BioEdit (www.mbio.ncsu.edu/BioEdit/bioedit.html) and compared for similarity to sequences available in GenBank (http://www.ncbi.nlm.nih.gov/BLAST).

**Table 1:** **Primers used for the amplification of DNA of** ***Babesia*** **spp.,** ***Hepatozoon*** **spp.,** ***Anaplasma*** **spp.,** ***Bartonella*** **spp** ***.,*** **‘** ***Candidatus*** **Neoehrlichia mikurensis’,** ***Ehrlichia canis*** **,** ***Rickettsia*** **spp. and filaroid nematodes**

**Specifity**	**Genetic marker**	**Sequences of primers (5’-3’)**	**Lenght of amplicons (bp)**	**Reference**
Apicomplexa	18S rRNA	**BTH-1 F**: CCT GAG AAA CGG CTA CCA CAT CT	561	[45]
		**BTH-1R**: TTG CGA CCA TAC TCC CCC CA		
*Babesia* spp.		Nested PCR:		
*Theileria* spp.		**GF2**: GTC TTG TAA TTG GAA TGA TGG		
		**GR2**: CCA AAG ACT TTG ATT TCT CTC		
*Hepatozoon canis*	18S rRNA	**H14Hepa18SFw**: GAA ATA ACA ATA CAA GGC AGT TAA AAT GCT	620	present study
		**H14Hepa18SRv**: GTG CTG AAG GAG TCG TTT ATA AAG A		
*Anaplasmataceae*	16S rRNA	**EHR16SD**: GGT ACC YAC AGA AGA AGT CC	345	[46]
		**EHR16SR**: TAG CAC TCA TCG TTT ACA GC		
*Bartonella* spp.	16S-23S rRNA	**bartg_for**: GAT GAT GAT CCC AAG CCT TC	134 - 315	modified [47]
		**B1623_rev**: AAC CAA CTG AGC TAC AAG CC		
*Rickettssia* spp.	23S/5S rRNA	**ITS-F**: GAT AGG TCG GGT GTG GAA G	342 - 533	[48]
		**ITS-R**: TCG GGA TGG GAT CGT GTG		
Filaroid nematodes	*COI*	**H14FilaCOIFw**: GCC TAT TTT GAT TGG TGG TTT TGG	724	present study
		**H14FilaCOIRv**: AGC AAT AAT CAT AGT AGC AGC ACT AA		

In order to detect apicomplexan parasites of the genera *Babesia* and *Hepatozoon*, the primer pair BTH-1 F and BTH-1R [45] was used. In those cases where the electropherograms showed superimposed signals, indicating mixed infections with different apicomplexan parasites, additional PCR reactions were performed with the specific primer pairs. The nested primers GF2 and GR2 [45] were used to detect *Babesia* spp., whereas new primers were designed for screening of *Hepatozoon* spp.: H14Hepa18SFw (5’- GAA ATA ACA ATA CAA GGC AGT TAA AAT GCT −3’) and H14Hepa18SRv (5A’- GTG CTG AAG GAG TCG TTT ATA AAG A −3’).

For PCR screening of spleens on the blood filaroid nematodes (e.g. *Dirofilaria*, *Acanthocheilonema*) another primer set, H14FilaCOIFw (5’- GCC TAT TTT GAT TGG TGG TTT TGG −3’) and H14FilaCOIRv (5’- AGC AAT AAT CAT AGT AGC AGC ACT AA −3’), was designed and used to amplify a 724 bp fragment of the mitochondrial cytochrome c oxidase subunit I (*COI*) gene (Table 1). The PCR mixture for the newly designed primer pairs was as follows: 1 μl of extracted DNA was added to 24 μl of reaction mixture containing 5 μl of 5X Green Reaction Buffer (7.5 mM MgCl_2_; pH 8.5), 0.5 μl of dNTPs (10 mM), 0.125 μl of Taq polymerase (5 u/μl), 2 μl of each primer (10 pmol/μl), and made up to a final volume of 25 μl with PCR grade water. Amplifications were conducted in a Mastercycler Pro (Eppendorf, Germany) under the following conditions: 95°C for 2 min followed by 35 cycles of 95°C for 60 s, 58°C (H14Hepa18S)/53°C (H14FilaCOI) for 60 s, 72°C for 60 s. Final extension was performed at 72°C for 5 min then held at 15°C.

### Statistical analyses

All statistical analyses were performed with SPSS 20.0 statistical software. The Kolmogorov-Smirnov test was used to test for normal distribution of the data. The Kruskal-Wallis test was chosen to compare proportions of positivity by geographical region, and the Mann–Whitney-U test was used to test pathogen distribution according to sex and age. Differences were considered significant at p < 0.05.

### Ethical statement

The study was conducted under the frame of Project ID: BIH-PSD-G-EC 30, Sub project ID: CRIS Number: 2010/022-259, for the improvement of animal health control through the vaccination against rabies and in accordance with the Veterinary law of Bosnia and Herzegovina.

## Results

**Figure 1:**
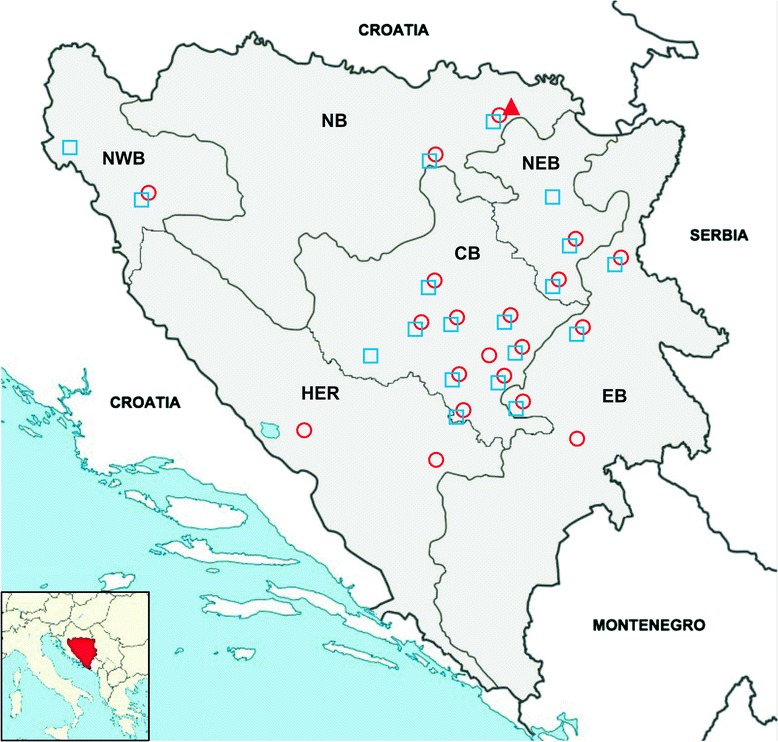
**Map of Bosnia and Herzegovina showing geographical locations of** ***Babesia canis***  **,** ***Babesia*** **cf.** ***microti***  **and** ***Hepatozoon canis***  **infected foxes.**

In the present study, three species of apicomplexan parasites, *B. canis*, *B.* cf. *microti* and *H. canis*, were identified in 73 red foxes by using molecular methods, with an overall prevalence of 60.8%. All infected foxes were in a good body condition and came from 23 municipalities of all six surveyed regions. The highest prevalence was detected in the region of North West Bosnia (Figure 1, Table 2). DNA of *B. canis*, *B.* cf. *microti* and *H. canis* was detected in 1 (0.8%; 95% confidence interval [CI]: 0.8–2.4%), 38 (31.9%; 95% CI: 23–40%) and 46 (38.6%; 95% CI: 30–48%) spleen samples, respectively. The geographical distribution of these pathogens overlap in many sampled areas (Figure 1) and co-infections with *B.* cf. *microti* and *H. canis* were confirmed in 11 animals (9.2%), while a single fox harboured all three pathogens (0.8%). There were no statistically significant differences in the prevalence of *B.* cf. *microti* infections between geographical regions, sex or age of the host. However, females (52.9%; 18/34) were significantly more infected with *H. canis* (p = 0.044) than males (32.9%; 28/85) (Table 3). Also, there was no statistically significant differences between the age and sex groups (p = 0.085).

**Table 2:** **The prevalence and geographical distribution of** ***B. canis*** **,** ***Babesia*** **cf.** ***microti*** **, and** ***Hepatozoon canis*** **infected foxes in Bosnia and Herzegovina**

**Region**	**No. of examined foxes**	***Babesia canis***		***Babesia*** **cf.** ***microti***		***Hepatozoon canis***		**Total infection** ^**A**^	
		**n**	**%**	**n**	**%**	**n**	**%**	**n**	**%**
East Bosnia (EB)	32	0	0.0	13	40.6	10	31.2	23	71.8
Central Bosnia (CB)	53	0	0.0	12	22.6	23	43.4	35	66.0
North Bosnia (NB)	9	1	11.1	2	22.2	3	33.3	6	66.6
Herzegovina (HER)	6	0	0.0	4	66.6	0	0.0	4	66.6
North East Bosnia (NEB)	15	0	0.0	6	40.0	7	46.6	13	86.6
North West Bosnia (NWB)	4	0	0.0	1	25.0	3	75.0	4	100
Total	119	1	0.8	38	31.9	46	38.3	85	70.8

^A^Refers to total number of infections, not total number of infected animals.

**Table 3:** **Number of foxes infected with** ***Babesia canis*** **,** ***Babesia*** **cf.** ***microti*** **and** ***Hepatozoon canis*** **in Bosnia and Herzegovina categorized by sex and age**

**Region**	***Babesia canis***				***Babesia*** **cf.** ***microti***				***Hepatozoon canis***			
	**Male**	**Female**	**>1 yr.**	**<1 yr.**	**Male**	**Female**	**>1 yr.**	**<1 yr.**	**Male**	**Female**	**>1 yr.**	**<1 yr.**
East Bosnia (EB)	-	-	-	-	6	7	13	-	5	5	10	-
Central Bosnia (CB)	-	-	-	-	8	4	10	2	14	9	22	1
North Bosnia (NB)	1	-	1	-	2	-	2	-	2	1	3	-
Herzegovina (HER)	-	-	-	-	4	-	2	2	-	-	-	-
North East Bosnia (NEB)	-	-	-	-	6	-	6	-	6	1	7	-
North West Bosnia (NWB)	-	-	-	-	1	-	1	-	1	2	3	-
Positive/Total sampled	1/85	0/34	1/112	0/7	27/85	11/34	34/112	4/7	28/85	18*/34	45/112	1/7

*(p < 0.05).

A total of 90 PCR-positive samples were sequenced. Of these, 38 samples showed 99–100% similarity to 18S sequences attributed to *Theileria annae* [GenBank accession no. HM212628.1] found in foxes from Croatia [3] and *Babesia* sp. ‘Spanish dog’ [GenBank accession no. AY457974.1] isolated from dogs in Spain [27]. A sequence of one sample showed 100% identity with sequences of *B. canis canis* [GenBank accession no. FJ209024.1] reported from dogs in Croatia [49].

PCRs performed with the Apicomplexa-specific 18S primers BTH-1 F and BTH-1R detected *H. canis* in 41 (89.1%) out of a total of 46 positive samples confirmed by *Hepatozoon*-specific PCR and sequencing. Moreover, five PCR products were positive on gel, but the occurrence of pathogens could not be confirmed by sequencing and all were noted as false positive.

Out of 46 18S sequences of *H. canis*, 38 were 100% identical to a sequence from a Spanish fox [GenBank accession no. AY150067.2] [38], while all others showed up to 99% similarity to sequences from foxes and dogs from all over the world (East Asia, India, Europe, North Africa and South America). All sequences are deposited in GenBank and are available under accession numbers KP216410–KP216494. The presence of *Anaplasma* spp., *Bartonella* spp., ‘*Candidatus* N. mikurensis’, *E. canis*, *Rickettsia* spp. and blood filaroid nematodes was not detected in our samples.

## Discussion

This study for the first time reports the occurrence of *B. canis*, *B.* cf. *microti* and *H. canis* parasites in red foxes from Bosnia and Herzegovina. The piroplasm *B.* cf. *microti* was molecularly confirmed in 38/119 (31.9%) animals from all six surveyed regions, with the highest prevalence (66.6%) detected in Herzegovina. The observed prevalence of infection was higher than that previously found in Croatia (5%; [3]), Italy (0.98%; [7]), Poland (0.7%; [4]) and Spain (14%; [30]). Higher prevalence was reported in foxes from Austria (50%; [29]), Germany (46.4%; [9]) and Portugal (69.2%; [6]). Differences between prevalence levels reported among studies may occur due to different tissues sampled or assay used, but also due to geographical distributions of the tick vectors [6]. *Ixodes hexagonus* was suspected to be the main vector responsible for transmission of *B.* cf. *microti* [50], but recently it was molecularly detected in *I. ricinus*, *I. canisuga* and *R. sanguineus* as well [9,51]. However, the vector competence of these tick species is not confirmed yet and the presence of the piroplasm DNA in the ticks may represent blood engorged from an infected animal host [6,9]. The fact that infected foxes were discovered in all studied areas, and that the occurrence of *I. hexagonus* was observed only in the western part of Bosnia [52], supports the hypothesis about the existence of another vector species other than *I. hexagonus*. Moreover, *B. canis* was detected in one fox (0.8%) originating from North Bosnia. This finding was completely unexpected, since *B. canis* has been molecularly confirmed only in a single fox from Portugal so far [6], suggesting that foxes are not suitable hosts for these canine blood parasites; they were probably transmitted accidentally by ticks which fed on infected dogs.

In this study, *H. canis* was the most frequently detected parasite, with a prevalence of 38.6% (46/119). Infected foxes were present in almost all sampled regions, except in the region of Herzegovina. This finding is intriguing because *R. sanguineus* is present in Herzegovina [52] and very small sample size obtained from this area (n = 6) may be the reason for the absence of *H. canis*. However, *H. canis* was also observed in areas lacking *R. sanguineus* [29,37,53]. Among studies using DNA from spleen or blood samples for PCRs, the prevalence of infections with *H. canis* in red foxes ranged from 8% in Hungary to 75.6% in Portugal [3,4,10,11,29-31,36,37]. The fact that there was no significant difference in the prevalence between the age groups of the host in our study might indicate that foxes were infected at a young age by vertical intrauterine transmission or by vectors as already suggested [10]. Since we had only 7 juveniles in our dataset, we cannot clearly confirm or reject the hypothesis of intrauterine transmission. But even though the age and sex groups were not statistically different, the samples represent not confounding data. Interestingly, the infection rate of females was significantly higher, which suggests that females have an important role in the maintaining and spreading of infection. It has been suggested that there is a difference in parasite burden between males and females and between parasitic taxa due to differences such as hormone level or innate immune response [54,55]. However, for *H. canis* the differences of the parasite load between females and males has not been explained, yet.

In dogs, infections with *B.* cf*. microti* and *H. canis* usually cause disorders that affect spleen, lymph nodes, bone marrow and kidneys, resulting in anemia, azotemia, fever, lethargy, cachexia or even death [25-28,35]. During necropsies it was noticed that all examined foxes, except for seven with sarcoptic mange (5.8%), were in a good body condition. This might indicate a low pathogenicity of these pathogens in this wild canid species, as already suggested [9]. All sequences obtained from red foxes in this study had a high homology to the ones previously reported from different canid species and different locations, which indicates a wide circulation of these pathogens without obvious geographical and host-related division patterns [31].

Although several studies suggest that red foxes can serve as reservoir hosts for various vector-borne bacteria [12-18], their presence could not be confirmed in this study. Regarding filaroid nematodes, *D. immitis* and *D. repens* were reported in dogs from Bosnia and Herzegovina [43], confirming that this area is suitable for the transmission of these parasites, but they also were not detected in foxes by PCR or necropsy. Since the present data does not allow the occurrence of bacteria and filaroid nematodes in foxes from Bosnia and Herzegovina to be completely excluded, monitoring and further analysis are necessary to elucidate the potential role of red foxes in their epidemiology.

## Conclusion

The relatively high prevalence and widespread distribution of *B.* cf. *microti* and *H. canis* among the red fox population of Bosnia and Herzegovina support the existence of a sylvatic cycle and reinforce the assumption that foxes might be a possible reservoir and vector of infection to dogs and other canids. Moreover, data presented in this study should improve awareness among veterinarians and alert them to include infections caused by these two pathogens in the differential diagnosis of canine babesiosis.
